# Is incidence of prostate cancer higher in patients with end stage renal disease?

**Published:** 2007

**Authors:** Makarand V. Khochikar

**Affiliations:** Department of Uro-oncology, Siddhi Vinayak Ganapati Cancer Hospital, Miraj, India. E-mail: khochikar@rocketmail.com

## SUMMARY

This paper is aimed at studying the usefulness of prostate-specific antigen (PSA) screening for patients with end-stage renal disease (ESRD) and also to assess if the incidence of prostate cancer is higher in this group of patients. The authors screened 1250 men with ESRD aged >50 years for prostate cancer and compared 1007 healthy men aged >55 years in the community who also underwent prostate cancer screening. Prostate-specific antigen > 4 ng/ml was taken as significant and further evaluation including prostate biopsy was done in these cases. The authors found a statistically significant increase in the PSA in the ESRD group than in healthy controls. Thirteen patients in the ESRD group and five in the healthy control men were found to have prostate cancer and were treated as per their staging. The authors conclude that higher levels of PSA are found in ESRD patients and there is higher incidence of prostate cancer in the ESRD group as compared to healthy men.

## COMMENTS

Though the issue of mass screening for prostate cancer in the general population is still a debatable one, reports of higher incidence of prostate cancer in ESRD patients[1] in the literature prompted this Japanese group to conduct a study to see if there is a higher incidence of prostate cancer in ESRD patients in the Japanese population and also to see the efficacy of PSA testing in these patients. There has been a concern over the PSA assay in the patients who are undergoing hemodialysis. Previous studies have shown that total PSA is not eliminated by hemodialysis and that the usual reference range for PSA can be used in these patients.[2]

The study is laudable not only for analyzing such a large number of patients (1250 with ESRD) but also for pursuing the possibility of malignancy in the patients undergoing hemodialysis. With advancement in the supportive care of ESRD more and more patients are living longer and there is no doubt that prevalence of malignant tumors, including prostate cancer is higher in patients with ESRD.

Authors have been successful in demonstrating the fact that PSA levels were higher and the incidence of prostate cancer was therefore also higher in these patients with ESRD in comparison to the healthy population. Looking at the treatments offered to these patients, some of the patients did undergo radical prostatectomy and radical radiation therapy for their early stage disease with no significant added morbidity.

However, the explanation given by the authors for higher levels of PSA in these groups is not truly satisfactory. They have suggested that lack of urinary symptoms in the presence oliguria, anuria perhaps do not prompt one to look at the possibility of prostatic cancer and therefore more patients present with higher PSA and higher stage. However, we all know that the urinary symptoms directly or indirectly have no bearing on the stage of the disease.
